# Integrative Taxonomy Supports Four New Species in the *Amphinemura sinensis* Species Group (Plecoptera: Nemouridae) from China †

**DOI:** 10.3390/insects17090909

**Published:** 2026-08-30

**Authors:** Yingying Wang, Guoquan Wang, Ding Yang, Weihai Li

**Affiliations:** 1 Guangxi Key Laboratory of Agric-Environment and Agric-Products Safety and National Demonstration Center for Experimental Plant Science Education, Agricultural College, Guangxi University, Nanning 530004, China; wyy5657@163.com; 2 Henan International Joint Laboratory of Taxonomy and Systematic Evolution of Insecta, Henan Institute of Science and Technology, Xinxiang 453003, China; 3 State Key Laboratory of Green Pesticides, Guizhou University, Guiyang 550025, China; dyangcau@126.com; 4 Observation and Research Station on Water Ecosystem in Danjiangkou Reservoir of Henan Province, Nanyang 474450, China

**Keywords:** stonefly, *Amphinemura sinensis* species group, molecular marker, new species, China

## Abstract

The *Amphinemura sinensis* species group is distributed in China and Vietnam and is characterized by the horn-shaped lateral processes of the dorsal sclerite and the pointed ventral sclerite of the male epiproct. Species in the group are often difficult to distinguish because of their similar morphology and the limited number of reliable diagnostic characters. In this study, we examined the *A. sinensis* species using morphological and molecular data. COI sequences were obtained for 16 of the 34 recognized species, including the four new species described herein. Detailed examination of male genital structures and comparisons with previously described species support the recognition of four new species. The four new species are described and illustrated. With these additions, the *A. sinensis* species group now comprises 34 species.

## Simple Summary

Stoneflies are common aquatic insects in mountain streams and are widely used in freshwater biomonitoring. Species of the *Amphinemura sinensis* species group are often difficult to distinguish based on external morphology because of their similar appearance. Accurate identification may require examination of fine morphological characters. In this study, we examined the *A. sinensis* species group using morphological and molecular data. COI DNA barcode sequences were obtained for 16 species, including the four new species described herein, representing approximately 47% of the 34 species recognized in the group. Based on morphological comparisons and molecular evidence, four new species are described. Our results increase the known diversity of the *A. sinensis* species group to 34 species and provide COI barcode data that can be used for species identification in future studies.

## 1. Introduction

*Amphinemura* is one of the most diverse genera of Nemouridae, comprising 225 known species distributed throughout the Holarctic and Oriental regions, including 30 species currently assigned to the *A. sinensis* species group [1]. Species of *Amphinemura* inhabit freshwater streams and are commonly found in mountain stream ecosystems. The *A. sinensis* species group was established primarily based on male genital characters, particularly the horn-shaped lateral processes of the dorsal sclerite and the pointed ventral sclerite of the male epiproct [2]. China represents an important center of diversity for *Amphinemura*, with 115 recorded species, 25 of which belong to the *A. sinensis* species group [3,4,5,6].

The *A. sinensis* species group has been the focus of continuous taxonomic work, especially in China, where most of the known diversity has been documented by Li et al. [2,7,8,9,10,11], Yang et al. [12,13], Mo et al. [4,14,15], Zhao et al. [3], and Zhao and Du [16]. However, species delimitation within the group remains difficult due to the high degree of morphological similarity among congeners. In particular, the diagnostic characters of the male terminalia are often subtle and show only minor interspecific variation, which can lead to uncertainty in species identification when based on limited material. In addition, many species are known from a small number of localities, and in some cases only from the type series, making it difficult to assess intraspecific variation and geographic distribution. As a result, further examination of newly collected material from different regions is essential for improving species-level taxonomy and understanding the diversity of the group.

During the examination of *Amphinemura* specimens from different localities in China, we recognized four species that do not correspond to any previously described species. These species are *Amphinemura coronata* Wang, Wang & Li, sp. nov., *Amphinemura gudoushan* Wang, Wang & Li, sp. nov., *Amphinemura quadrispina* Wang, Wang & Li, sp. nov., *Amphinemura prominens* Wang, Wang & Li, sp. nov. The four species were collected from southern China, increasing the number of recognized species in the *A. sinensis* species group from 30 to 34.

In this study, we examine the *A. sinensis* species group based on morphological examination and COI DNA barcode data. Detailed descriptions, diagnoses, illustrations, and photographs are provided for the four new species based on morphological examination of adult males and females. Larvae are unknown. A maximum likelihood analysis was conducted to examine the relationships among the sampled species. The molecular results are compared with the morphological characters used to distinguish the species.

## 2. Materials and Methods

### 2.1. Sample Collection and Morphological Study

All adults were collected by handpicking, sweeping, Malaise trap, or light trapping and preserved in 95% ethanol. The male terminalia were cleared in 10% NaOH. The types are deposited in the Natural History Museum of China (NHMC) and the Insect Collection of Henan Institute of Science and Technology, Xinxiang (HIST). Specimens for molecular analysis were stored at −20 °C.

Specimens were examined under a Leica S8AP0 stereomicroscope. Color photographs were taken using a Leica M205 FCA stereomicroscope equipped with a digital camera. Morphological terminology follows Baumann [17] and Mo et al. [4].

### 2.2. DNA Extraction, PCR Amplification, and Sequencing

Genomic DNA was extracted from leg muscle tissues or thoracic tissues using the TIANamp Micro DNA Kit (Tiangen Biotechnology, Beijing, China) following the manufacturer’s protocol. A fragment of the mitochondrial cytochrome c oxidase subunit I (COI) gene was amplified using the primers LCO1490 (5′-GGTCWACWAATCATAAAGATATTGG-3) and HCO2198 (5′-TAAACTTCWGGRTGWCAAARAATCA-3′).

PCR reactions were performed in a total volume of 25 μL containing 14.5 μL of ultrapure water, 5 μL of Taq buffer, 1 μL of each primer (0.01 μM), 2 μL of each dNTP (0.05 mM), 0.5 μL of Easy Taq polymerase, and 1 μL of DNA template. The amplification conditions were as follows: initial denaturation at 95 °C for 30 s; 40 cycles of denaturation at 95 °C for 30 s, annealing at 40–60 °C for 50 s, and extension at 65 °C for 60 s; followed by a final extension at 65 °C for 10 min. PCR products were checked by electrophoresis on 1% agarose gels. Successful amplicons were purified and bidirectionally sequenced using the Sanger method by Sangon Biotech (Shanghai, China). Newly generated COI sequences were deposited in GenBank, and accession numbers are provided in Table 1. Phylogenetic analyses included the newly generated COI sequences and two outgroup sequences retrieved from GenBank.

### 2.3. Phylogenetic Analyses

Two sequences were retrieved from NCBI GenBank and used as outgroups: *Capnia oblata* Chen and Du, 2017 (PQ559514) and *Neoperla baishuijiangensis* Du, 2005 (PX504994). Phylogenetic relationships were inferred using maximum likelihood (ML) in IQ-TREE [18]. The best-fit substitution model was selected using ModelFinder based on the Bayesian information criterion (BIC), and TVM+F+I+G4 was selected as the best fit model. Branch support was assessed using 1000 ultrafast bootstrap replicates. The resulting ML tree was visualized and annotated using iTOL [19]. Pairwise genetic distances among the COI sequences were calculated under the Kimura 2-parameter (K2P) model [20] using MEGA v. 11 [21].

## 3. Results

### 3.1. Molecular Phylogeny

**Figure 1:**
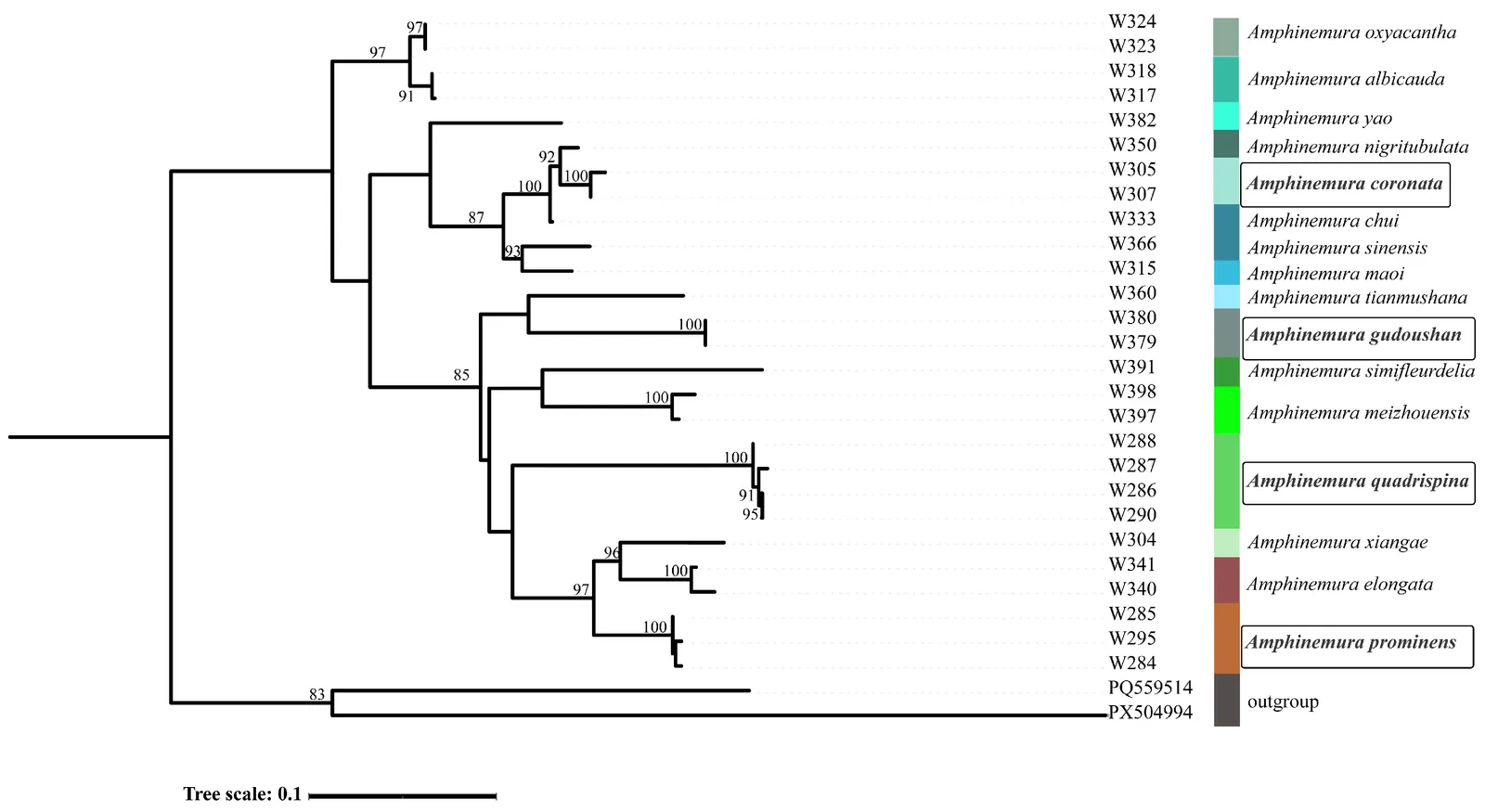
Maximum likelihood (ML) tree of the *Amphinemura sinensis* species group based on COI sequences under the TVM+F+I+G4 model. Numbers at the nodes indicate bootstrap support values. The scale bar indicates the genetic distance.

The final COI alignment comprised 29 sequences, each 658 bp in length, including 27 ingroup and two outgroup sequences. The resulting tree recovered most species as distinct clusters, with moderate to strong bootstrap support (Figure 1).

The four newly described species, *A. coronata*, *A. quadrispina*, *A. prominens*, and *A. gudoushan*, each formed a distinct clade in the ML tree. *Amphinemura coronata* was recovered with strong support (BS = 100), as were *A. quadrispina* (BS = 95), *A. prominens* (BS = 100), and *A. gudoushan* (BS = 100). Sequences within each species showed low genetic divergence, whereas genetic distances among most morphologically distinct species were higher (Table S1).

Pairwise genetic distances were calculated using the Kimura 2-parameter (K2P) model. Intraspecific distances ranged from 0.00 to 0.016, whereas interspecific distances ranged from 0.013 to 0.192 (Table S1). The four newly described species were also separated from their sampled congeners in the COI phylogeny, providing additional molecular support for their morphological recognition.

### 3.2. Taxonomy

*Nemura* [sic] (*Amphinemura*) Ris, 1902: 384. [22].

*Nemura* [sic] (*Amphinemura*) Ris, 1902 in Klapálek, 1909: 75. [23]. Type-species: *Nemoura cinerea* Olivier, 1811 [= *Amphinemura sulcicollis* (Stephens, 1835)].

**Diagnosis.** Small to medium-sized. Cervical gills forked, with two branches on each side. Paraproct trilobed.

**Distribution.** Holarctic and Oriental [1].

#### 3.2.1. *Amphinemura coronata* Wang, Wang & Li, sp. nov.

**Figure 2:**
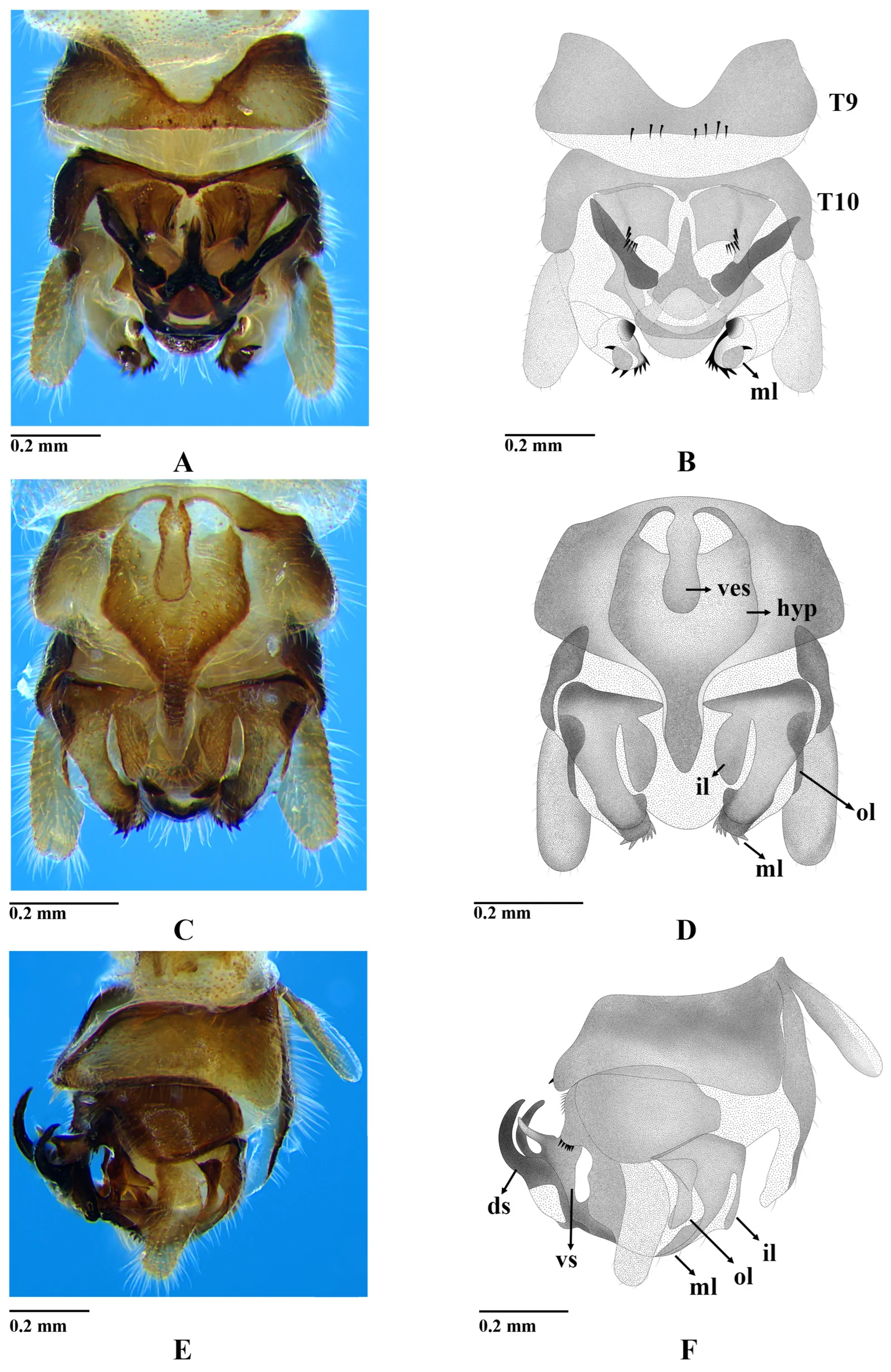
*Amphinemura coronata* sp. nov., male terminalia. (**A**,**B**) Dorsal view; (**C**,**D**) ventral view; (**E**,**F**) lateral view. Abbreviations: T9, tergum 9; T10, tergum 10; ds, dorsal sclerite; vs, ventral sclerite; ves, vesicle; hyp, hypoproct; il, inner lobe; ml, median lobe; ol, outer lobe.

**Figure 3:**
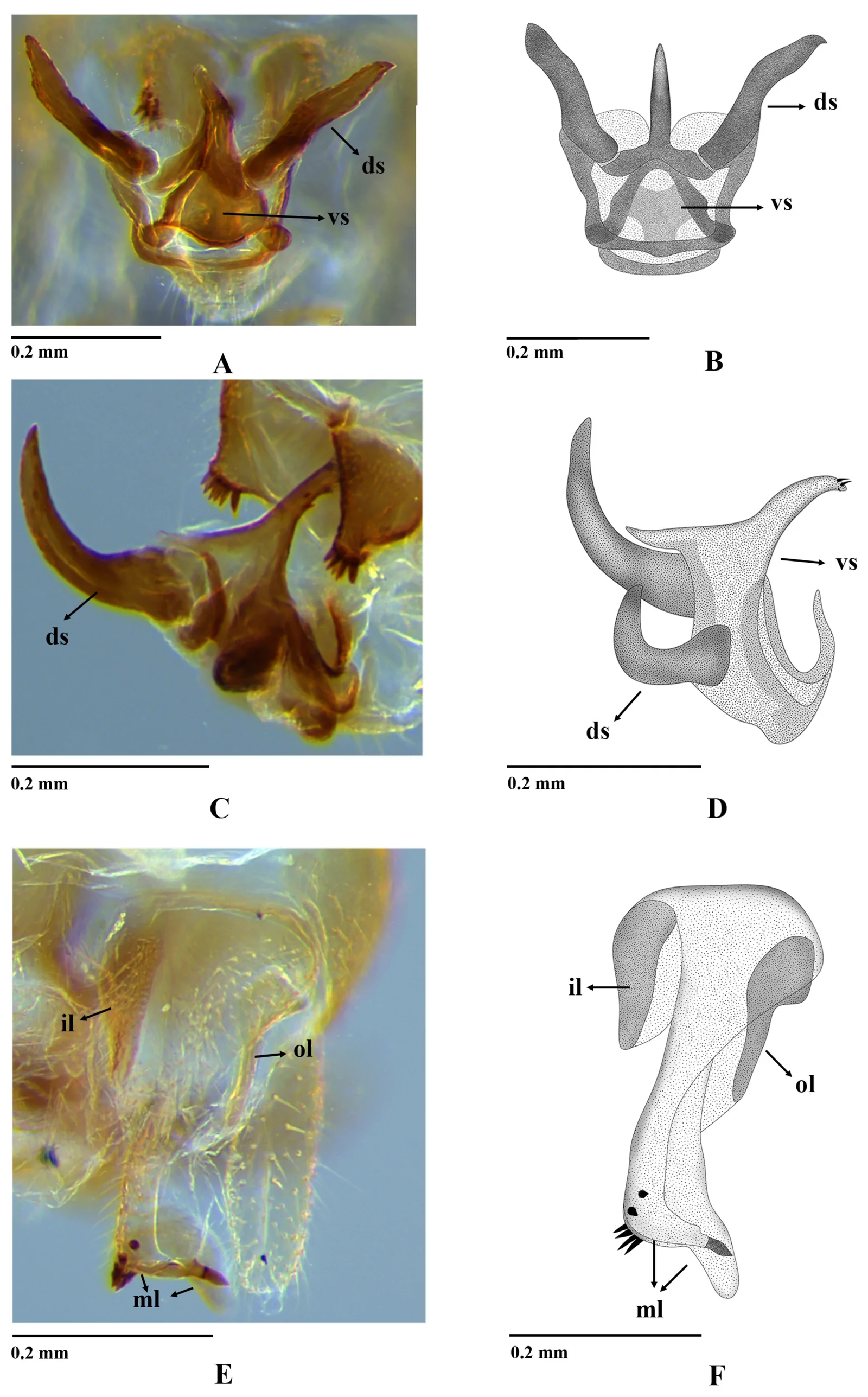
*Amphinemura coronata* sp. nov., male. (**A**–**D**) Epiproct: (**A**,**B**) dorsal view; (**C**,**D**) lateral view; (**E**,**F**) paraproct, caudal view. Abbreviations: ds, dorsal sclerite; vs, ventral sclerite; il, inner lobe; ml, median lobe; ol, outer lobe.

**Figure 4:**
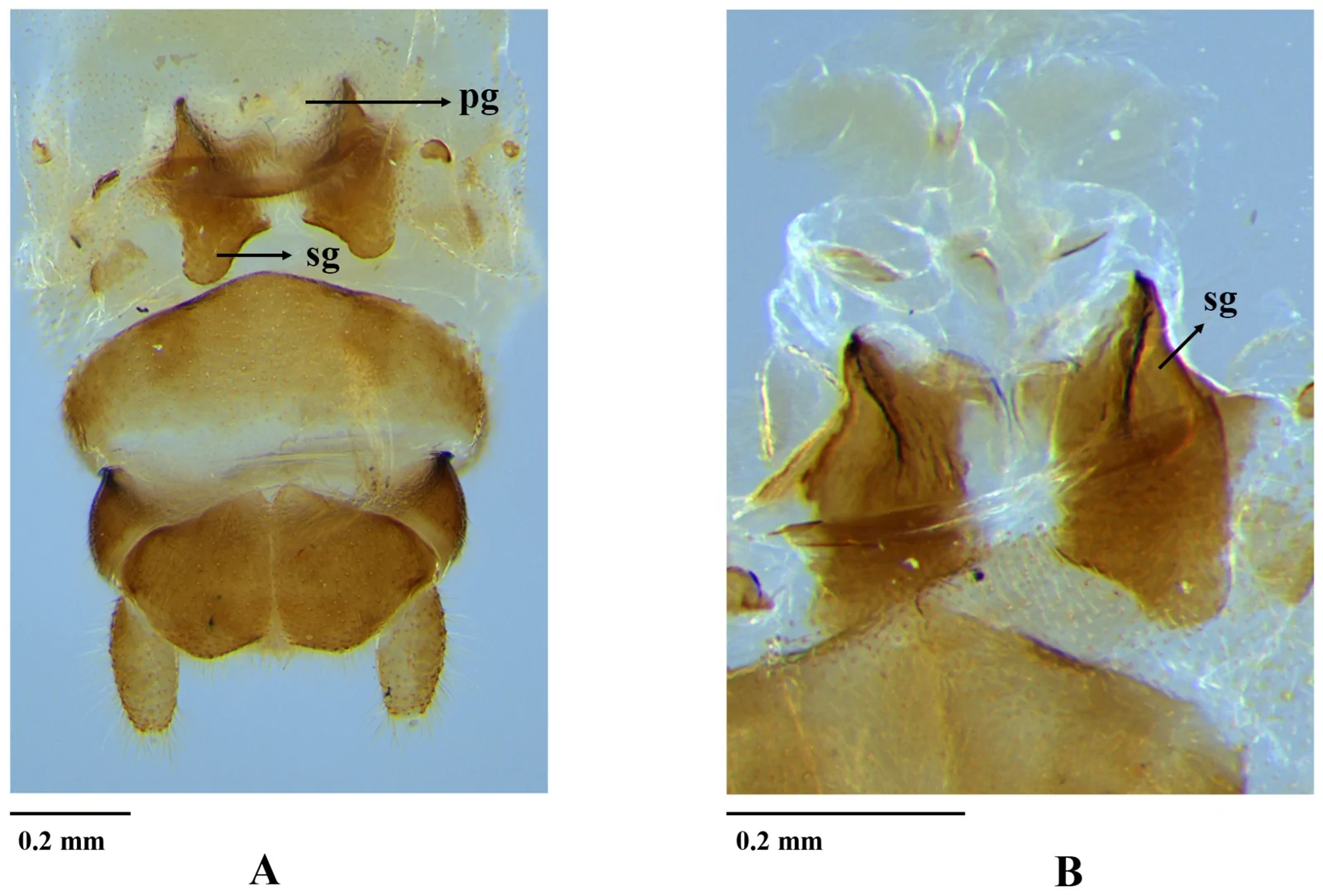
*Amphinemura coronata* sp. nov, female. (**A**) Terminalia after being cleared, ventral view; (**B**) inner genitalia, ventral view. Abbreviations: pg: pregenital plate; sg: subgenital plate.

Chinese common name: 冠状倍叉𧐐

(Figure 2, Figure 3 and Figure 4)

**Material examined. Holotype.** ♂ (HIST), China, Guizhou Province, Chishui City, Alsophila National Nature Reserve, Foguangyan, 451 m, 28°24′09″ N, 105°56′53″ E, 1-IV-2012, Weihai Li leg. **Paratypes**. 1♂2♀ (HIST), same data as holotype; 5♂5♀ (NHMC), 9♂7♀ (HIST), China, Guizhou Province, Zunyi City, Xishui County, Sancha River, 1161 m, 28°29′52″ N, 106°23′55″ E, 30-III-2012, Weihai Li leg.

**Diagnosis. ***Amphinemura coronata* is distinguished from congeners by a crown-like dorsal sclerite, and a paraproctal median lobe bearing two median spines on the upper lobe and four spines along the inner margin of the lower lobe.

**Description.**

**Male** (Figure 2 and Figure 3). Forewing length 5.3–5.8 mm, hindwing length 4.4–4.6 mm (*n* = 2). General body brown. Head dark brown; compound eyes black; antennae dark brown; head wider than pronotum. Pronotum subquadrate, dark brown. Legs light brown. Wing membranes subhyaline, veins brown. Abdominal segments brown, with darker terminalia.

Tergum IX (Figure 2) slightly sclerotized, anterior margin concave; with a middle trapezoidal sclerite and posterior covered with several black spines (Figure 2A,B). Vesicle of sternum IX (Figure 2C,D) claviform, approximately 3.5× as long as maximum width. Hypoproct pentagonal basally, gradually tapering into a membranous, incurved apex (Figure 2C,D). Tergum X (Figure 2) strongly sclerotized, with a membranous concavity beneath the epiproct and a pair of short paramedial black spines. Cercus (Figure 2) cylindrical, about 3× as long as maximum width (Figure 2A,B).

Epiproct about 2× wider than long, distal portion trifurcate with median process membranous (Figure 2A,B and Figure 3A–D). Distal portion of dorsal sclerite with pair of strong black lateral arms curved outward; basal and median portions mostly membranous. Ventral sclerite strongly sclerotized, laterally expanded into a triangular ridge; broad at base, tapering to an incurved sclerotized apex with several spines; the apex additionally bearing an outcurved, pointed, sclerotized finger-like process. 

Paraproct (Figure 2C,D and Figure 3E,F) trilobed: inner lobe triangular, strongly sclerotized, bearing hairs; median lobe large, with a bifurcate apex; upper bearing two median spines and a black apical spine, lower lobe mostly membranous, with four short black spines subapically along the inner margin; apex strongly sclerotized and finger-like; outer lobe strongly sclerotized basally, with a fine apical projection.

**Female** (Figure 4). Forewing length 5.7–6.1 mm, hindwing length 4.4–5.2 mm (*n* = 5). Sternum VII produced into a large, broadly transverse, sclerotized pregenital plate, posterior margin broadly rounded, covering the anterior half of sternum VIII and the inner sclerite (Figure 4). Sternum VIII forms subtriangular, pale subgenital plate, posterior margin slightly concave medially, bearing a pair of strongly sclerotized, dark posterolateral sclerites clearly visible under transmitted light (Figure 4). Paragenital plates paired, forming brown, subtriangular lobes near lateral margins of subgenital plate (Figure 4). Paraprocts wide and subrectangular, with obtuse apices. Inner genitalia (Figure 4B) mostly membranous, rectangular, with paired small, sclerotized structures, connected to the spermathecal ductus.

**Etymology.** The specific epithet ‘coronata’ is derived from Latin ‘coronatus’ (crowned), referring to the crown-shaped dorsal sclerite of the epiproct. 

**Distribution.** China (Guizhou Province). 

**Remarks**. The new species is similar to *A. chui* Wu, 1935, described from Guangdong and Zhejiang Provinces, in the general paraproctal structure. However, *A. coronata* can be distinguished from *A. chui* by the strong black lateral arms, the median projection presented as crown which is unique in this group, and the ventral sclerite bearing a spinose apical process, which is absent in *A. chui* [16]. Furthermore, the paraproctal median lobe of *A. coronata* bears two median spines on the upper lobe and four spines along the inner margin of the lower lobe. In contrast, the upper lobe of the median lobe in *A. chui* bears a distal row of 4–6 stout black spines, while the lower lobe is lightly sclerotized and acuminate [16]. The female resembles that of *A. chui*, while in *A. chui*, there is a longitudinal band between the paired subgenital plates which is absent in the new species; their distinctness is also corroborated by male COI data (Figures 4 and 5B,C in [16]).

#### 3.2.2. *Amphinemura quadrispina* Wang, Wang & Li, sp. nov.

**Figure 5:**
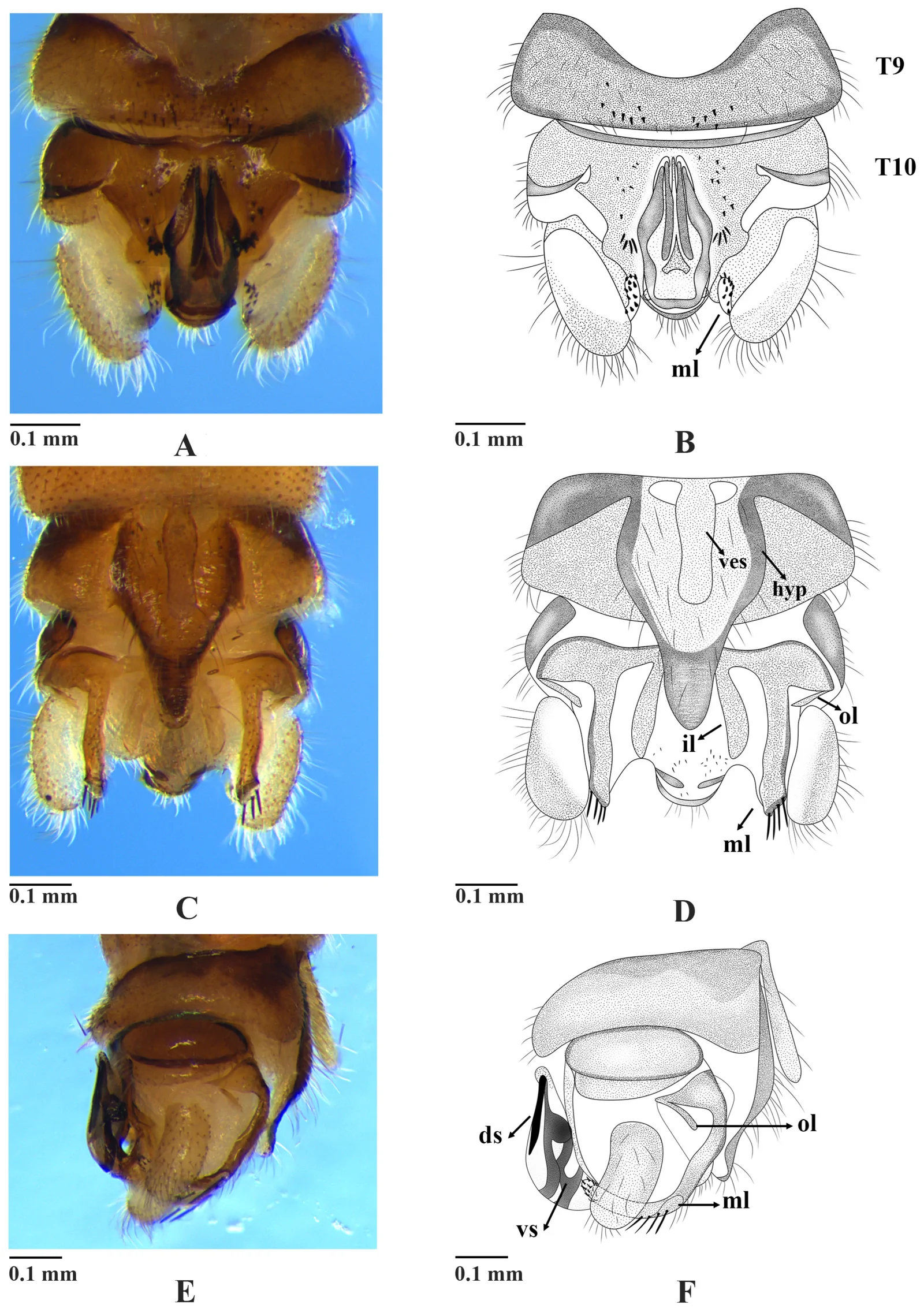
*Amphinemura quadrispina* sp. nov., male terminalia. (**A**,**B**) Dorsal view; (**C**,**D**) ventral view; (**E**,**F**) lateral view. Abbreviations: T9, tergum 9; T10, tergum 10; ds, dorsal sclerite; vs, ventral sclerite; ves, vesicle; hyp, hypoproct; il, inner lobe; ml, median lobe; ol, outer lobe.

**Figure 6:**
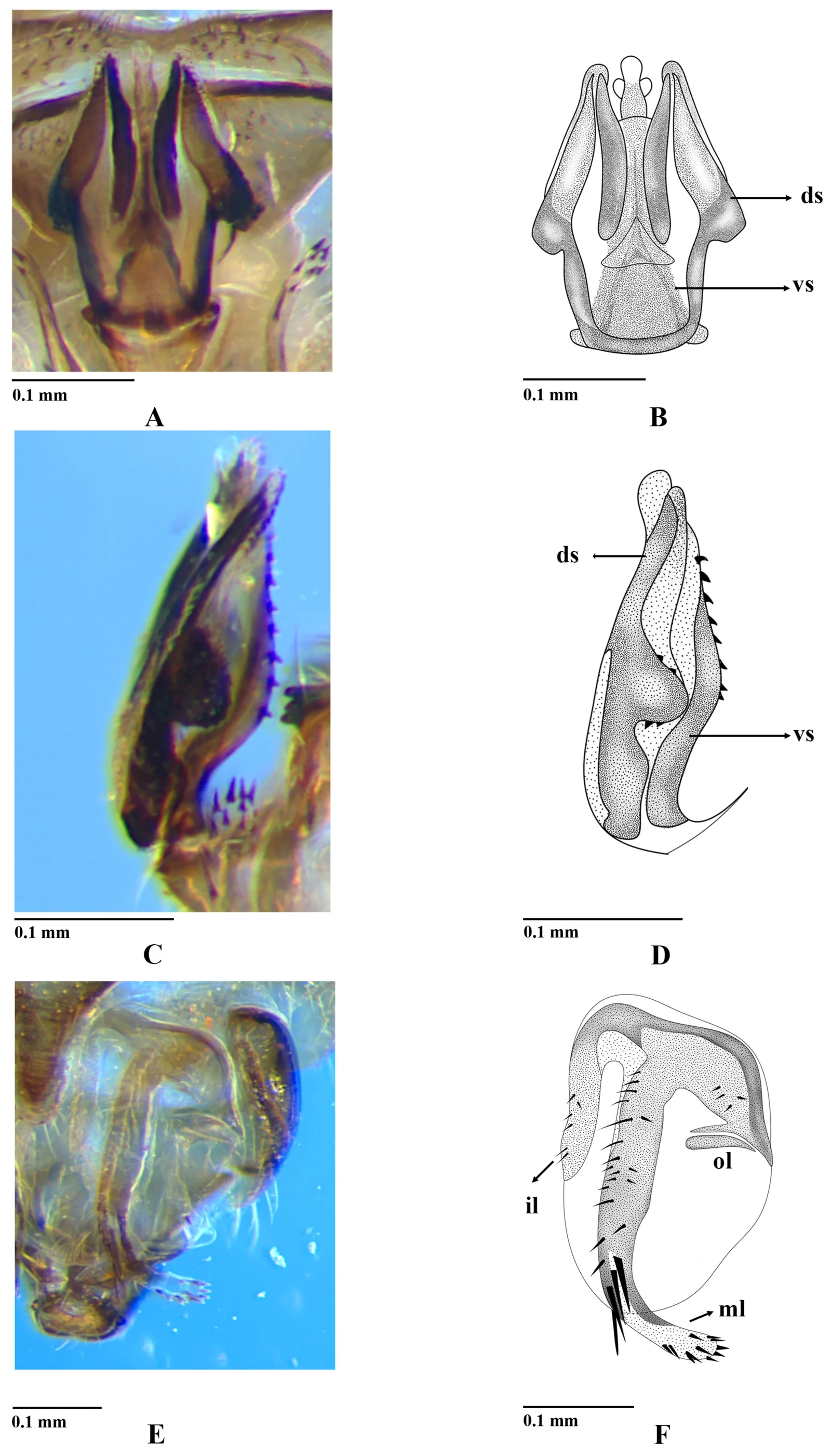
*Amphinemura quadrispina* sp. nov., male. (**A**–**D**) Epiproct: (**A**,**B**) dorsal view; (**C**,**D**) lateral view; (**E**,**F**) paraproct, caudal view. Abbreviations: ds, dorsal sclerite; vs, ventral sclerite; il, inner lobe; ml, median lobe; ol, outer lobe.

**Figure 7:**
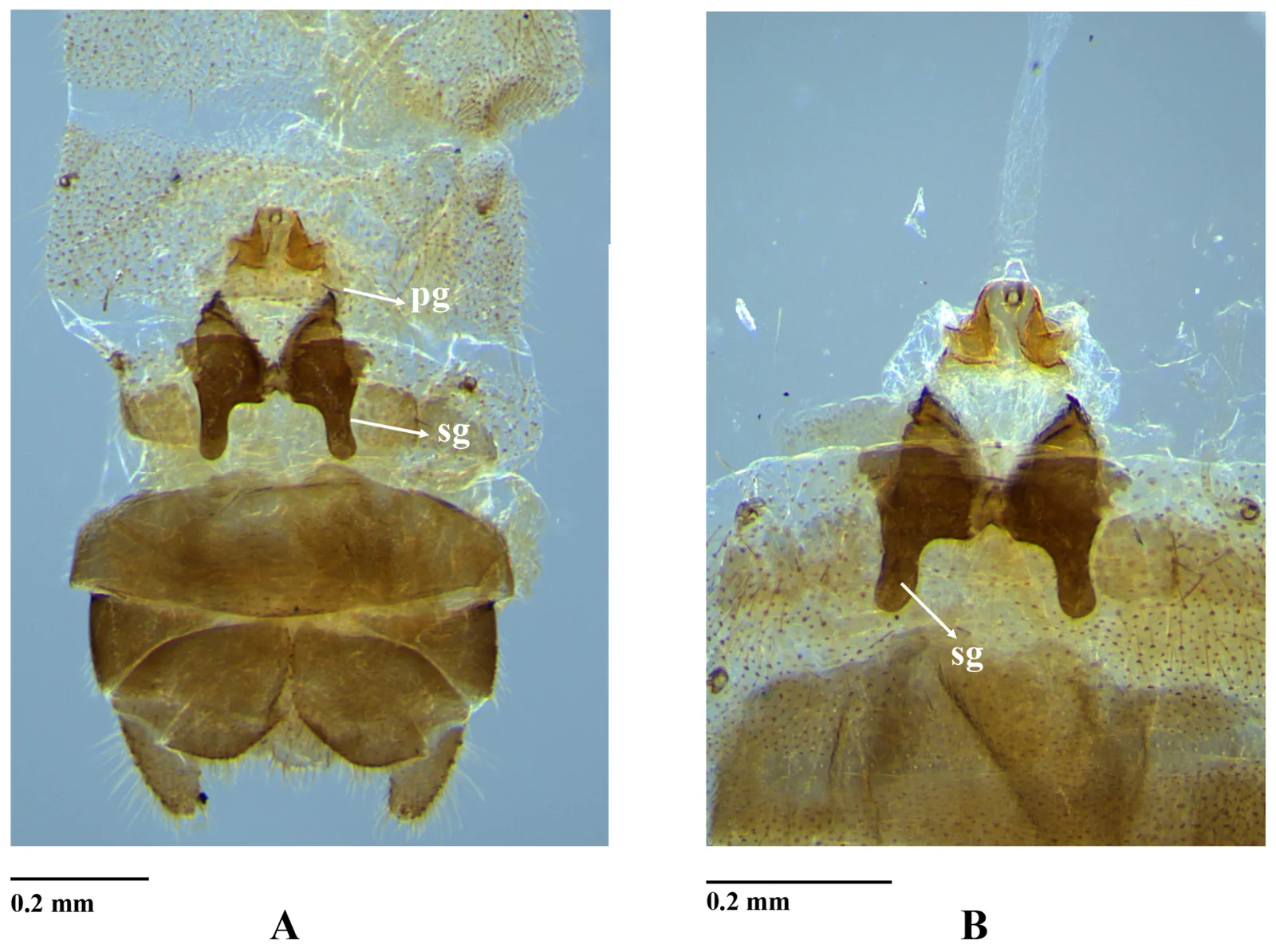
*Amphinemura quadrispina* sp. nov., female. (**A**) Terminalia after being cleared, ventral view; (**B**) inner genitalia, ventral view. Abbreviations: pg: pregenital plate; sg: subgenital plate.

Chinese common name: 四刺倍叉𧐐

(Figure 5, Figure 6 and Figure 7)

**Material examined. Holotype.** ♂ (HIST), China, Guizhou Province, Qiandongnan Miao and Dong Autonomous Prefecture, Leishan County, Leigong Mountain National Nature Reserve, Leigong Mountain Station, 1595 m, 26°21′44″ N, 108°09′39″ E, 24-VI-2019, Malaise trap, Tao Li leg. **Paratypes.** 1♀ (HIST), same data as holotype; 6♂4♀ (NHMC), China, Guangxi Zhuang Autonomous Region, Liuzhou City, Rongshui County, Qingshuitan Conservation Station, 365 m, 25°11′58″ N, 108°47′45″ E, 7-V-2024, Light, Yingying Wang leg.

**Diagnosis. ***Amphinemura quadrispina* differs from congeners by the epiproct with sclerotized lateral arms and spiny, black triangular sclerites, and by the L-shaped paraproctal median lobe bearing four black spines and two to three rows of stout apical spines.

**Description.**

**Adult habitus.** Body brown. Head mostly dark brown; compound eyes black; antennae mostly brown, with scape and pedicel pale; palpi brown. Head wider than pronotum; pronotum subquadrate. Legs brown, tibia brownish, tarsi dark brown. Wing membranes subhyaline, veins brown. Abdominal segments brown, with dark brown terminalia.

**Male** (Figure 5 and Figure 6). Forewing length 5.8–6.4 mm, hindwing length 4.7–5.5 mm (*n* = 6). Tergum IX (Figure 5A,B) strongly sclerotized, with anterior margin emarginated, with two paramedial patches of spinules, and posterior covered with several bristles. Sternum IX with claviform vesicle (Figure 5C,D), approximately 2.5× as long as maximum width. Hypoproct broad basally and tapering to apex with several setae. Tergum X (Figure 5A,B,E,F) strongly sclerotized, with light brown concavity beneath the epiproct, with two groups of black spines medially and sparse spinules. Cercus (Figure 5) cylindrical, slightly sclerotized laterally, about 2× as long as maximum width.

Epiproct nearly triangular, about 1.5× wider than long, with distal portion trifurcate. Dorsal sclerite with a pair of sclerotized lateral arms and black triangular sclerites (Figure 6A–D). Ventral sclerite brown and subtriangular, broad at base, tapering to the tip and forming a triangular ridge bearing a row of tiny black spines. 

Paraproct (Figure 5C,D and Figure 6E,F) brown, trilobed: inner lobe sclerotized, nearly triangular, with obtuse and outward-curved apex; median lobe L-shaped, bearing four black spines subapically on ventral surface, apex strongly curved upward, armed with 2–3 rows of stout apical spines; outer lobe transverse, narrow, strongly sclerotized, and distinctly incurved apically.

**Female** (Figure 7). Forewing length 5.8–6.1 mm, hindwing length 4.6–4.9 mm (*n* = 5). Sternum VII produced into a small semicircular pregenital plate, weakly sclerotized (Figure 7A,B). Sternum VIII forming a sclerotized, rectangular subgenital plate, with a finger-like apex. Paragenital plates paired, forming small and rectangular brownish patches connected with posterolateral margin of subgenital plate. Sternum IX trapezoidal, median half protruded anteriorly, in ventral aspect without anterior indentation. Paraproct brown, apex rounded widely. Inner genitalia (Figure 7B) mostly membranous, triangular, with a trapezoidal sclerotized structure, apical cone membranous with an apical circular sclerite ring, connected to the spermathecal ductus.

**Etymology.** The specific name ‘quadrispina’ is a noun in apposition, derived from the Latin ‘quadri-’(four) and ‘spina’ (spine), referring to the four long black spines on the subapical area of the median lobe of the paraproct.

**Distribution.** China (Guizhou Province and Guangxi Zhuang Autonomous Region).

**Remarks**. It is most similar to *Amphinemura meizhouensis* Mo, Wang, Yang, Li and Murányi, 2021, described from Guangdong Province, in having a similar dorsal sclerite of epiproct and the male terminalia [15]. However, *A. quadrispina* can be easily distinguished from *A. meizhouensis* by the median lobe of the paraproct bearing four long black subapical spines and several stout spines at the apex. The female is similar to *Amphinemura xiangae* Zhao, Du and Rehman, 2022, but the two species can be distinguished by molecular evidence, with the COI sequences supporting their separation [3].

#### 3.2.3. *Amphinemura gudoushan* Wang, Wang & Li, sp. nov.

**Figure 8:**
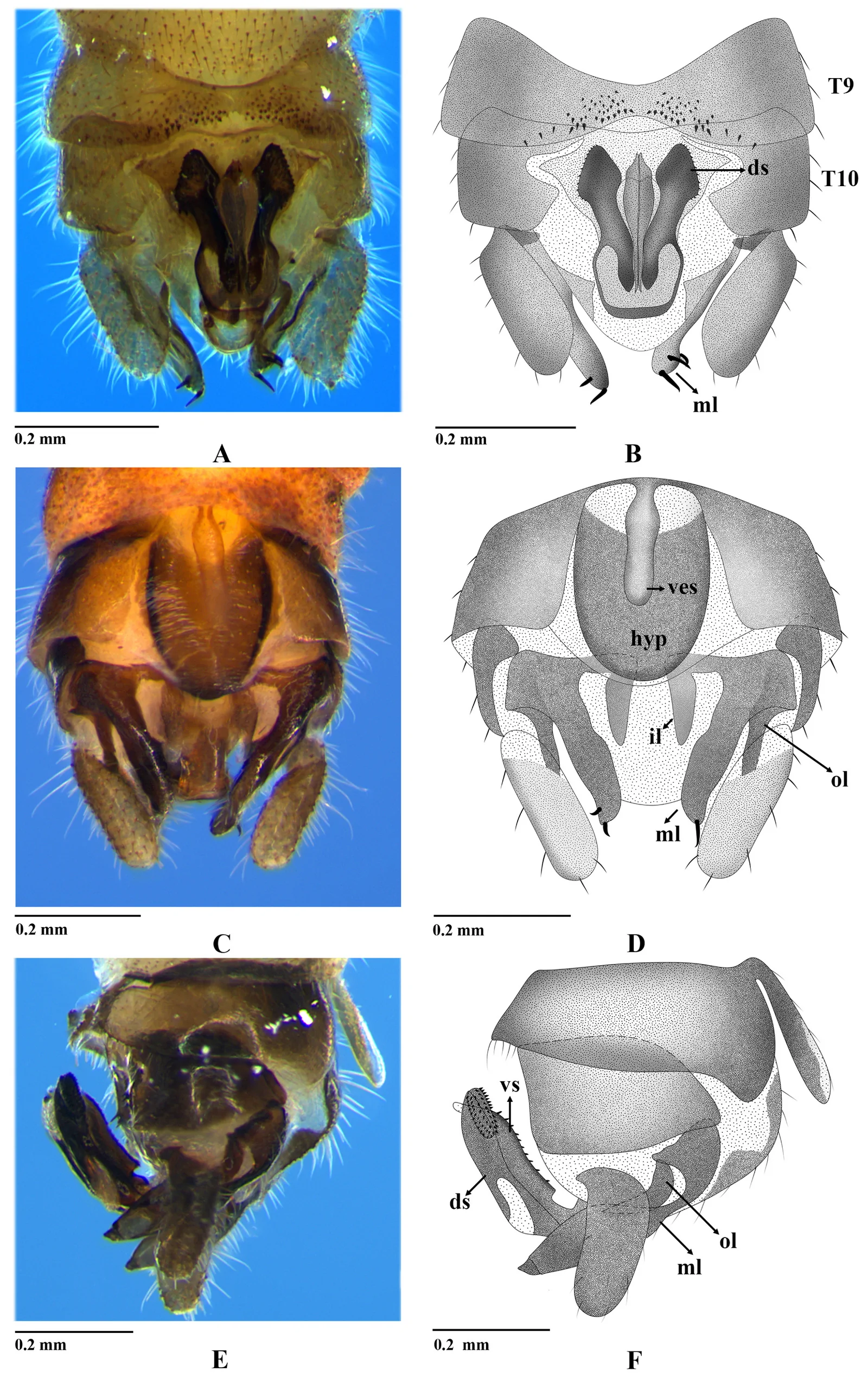
*Amphinemura gudoushan* sp. nov., male terminalia. (**A**,**B**) Dorsal view; (**C**,**D**) ventral view; (**E**,**F**) lateral view. Abbreviations: T9, tergum 9; T10, tergum 10; ds, dorsal sclerite; vs, ventral sclerite; ves, vesicle; hyp, hypoproct; il, inner lobe; ml, median lobe; ol, outer lobe.

**Figure 9:**
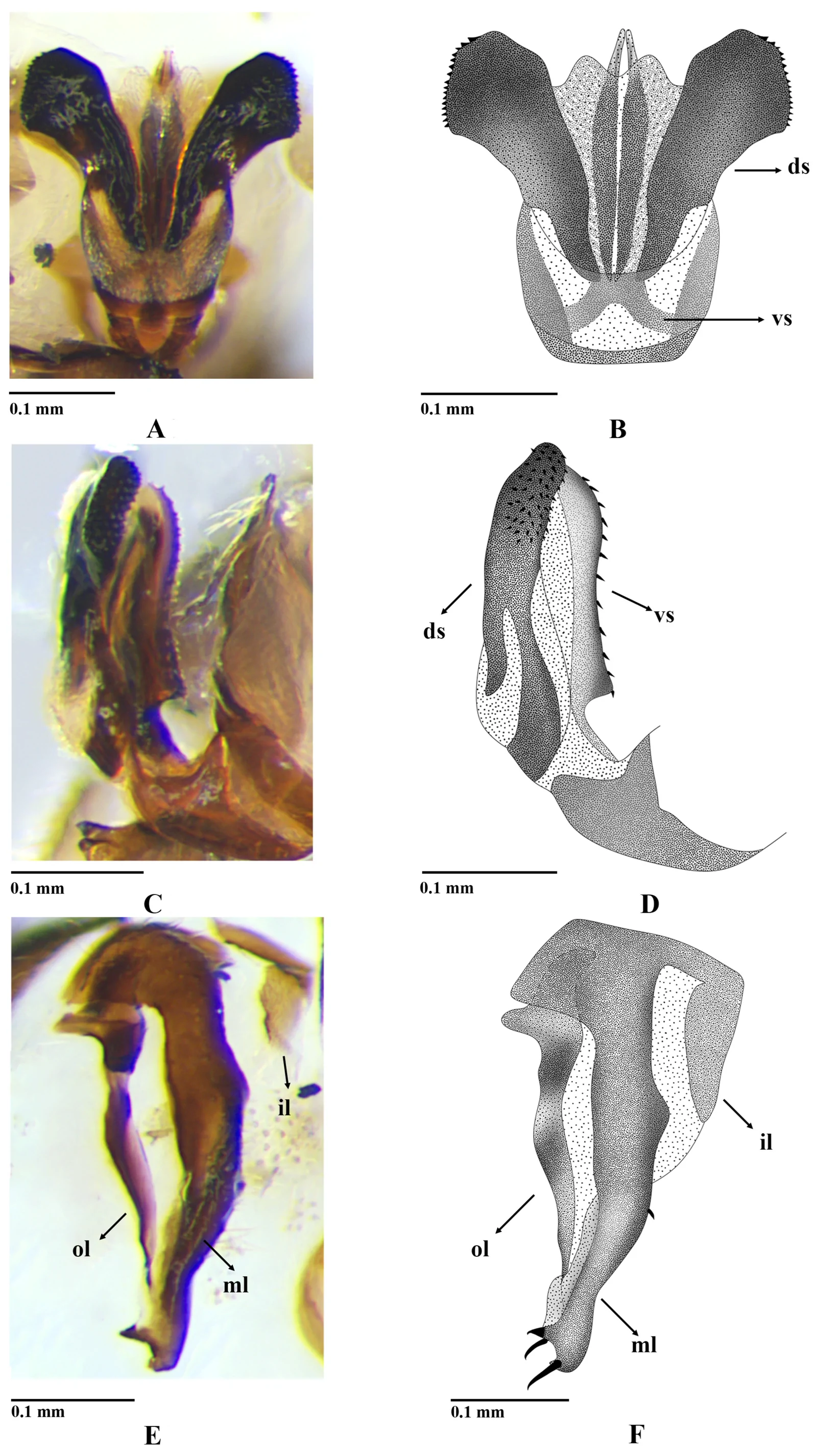
*Amphinemura gudoushan* sp. nov., male. (**A**–**D**) Epiproct: (**A**,**B**) dorsal view; (**C**,**D**) lateral view; (**E**,**F**) paraproct, caudal view. Abbreviations: ds, dorsal sclerite; vs, ventral sclerite; il, inner lobe; ml, median lobe; ol, outer lobe.

**Figure 10:**
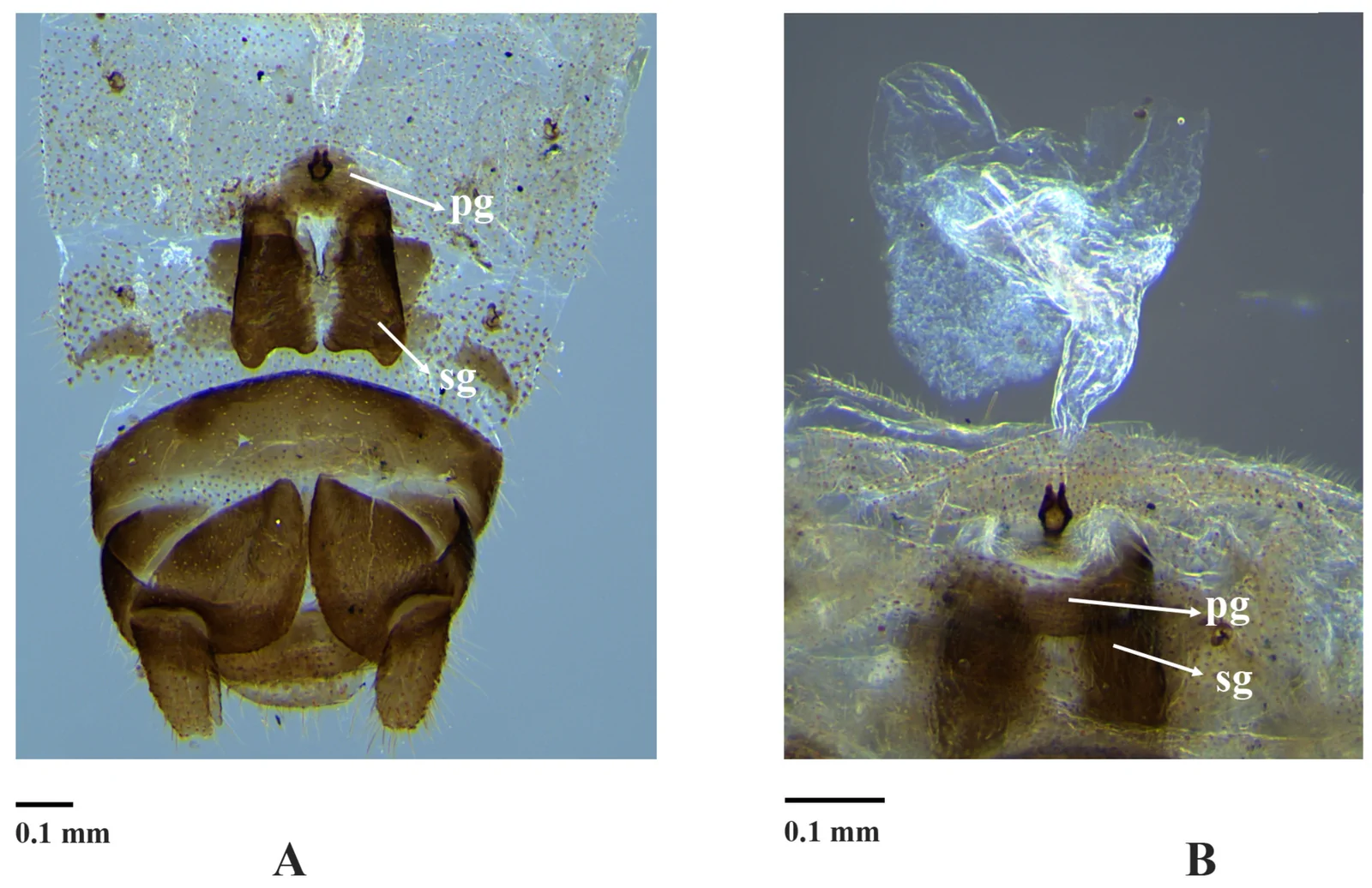
*Amphinemura gudoushan* sp. nov., female. (**A**) Terminalia after being cleared, ventral view; (**B**) inner genitalia, ventral view. Abbreviations: pg: pregenital plate; sg: subgenital plate.

Chinese common name: 古兜山倍叉𧐐

(Figure 8, Figure 9 and Figure 10)

**Material examined. Holotype.** ♂ (HIST), China, Guangdong Province, Jiangmen City, Gudoushan Provincial Nature Reserve, Malaise trap, 539 m, 22°14′03″ N, 112°56′57″ E, 24.I.2024–18.IV.2024. **Paratypes**. 1♂2♀ (HIST), 4♀ (NHMC) same data as holotype; 3♂3♀ (NHMC), China, Guangdong Province, Jiangmen City, Gudoushan Provincial Nature Reserve, Malaise trap, 339 m, 22°13′10″ N, 112°56′4″ E, 2023.XI.29-2024.I.24.

**Diagnosis. ***Amphinemura gudoushan* is distinguished from congeners in having an epiproct with a spiny, arrowhead-like tip, and a median lobe bearing two subapical spines and one black apical spine on the dorsal surface.

**Description.**

**Adult habitus.** General body brown. Head dark brown; antennae mostly dark brown, palpi paler; head wider than pronotum; pronotum subquadrate; legs brown, tarsi darker. Wing membranes infuscate, veins dark brown. Abdominal segments brown with dark brown terminalia.

**Male**. (Figure 8 and Figure 9). Forewing length 5.1–5.2 mm, hindwing length 3.9–4.2 mm (*n* = 2). Tergum IX (Figure 8) strongly sclerotized, with a pair of spinous patches present along posterior margin. Tergum X (Figure 8A,B) strongly sclerotized, with membranous concavity beneath the epiproct, two clusters of short black spines present on side of the sclerite. Vesicle of sternum IX claviform, approximately 3× as long as maximum width (Figure 8C,D). Hypoproct subrectangular, mostly sclerotized except for a membranous basal area, broad basally and gradually tapering toward apex (Figure 8C,D). Cercus cylindrical, about 2× as long as maximum width (Figure 8).

Epiproct about 0.5× wider than long, distal portion trifurcate (Figure 8A,B and Figure 9A,B). Dorsal sclerite with pair of broad lateral arms curved outward and extending toward the apex, with arrowhead-like tip; outer margins covered with tiny black spines. Ventral sclerite dark brown and strongly sclerotized, broad basally, gradually tapering and converging toward apex, forming a triangular ridge bearing rows of 24–26 short black spines.

Paraproct (Figure 8C,D and Figure 9C,D) trilobed: inner lobe triangular, weakly sclerotized, with obtuse apex; median lobe strongly sclerotized, bearing two subapical spines and one black apical spine on the dorsal surface, with the apex strongly incurved; outer lobe triangular and slender, with a darkly sclerotized outer margin.

**Female** (Figure 10). Forewing length 5.8–6.1 mm, hindwing length 4.6–4.9 mm (*n* = 3). Female. Sternum VII produced into a large, broadly semicircular, sclerotized pregenital plate, posterior margin broadly rounded and slightly concave, covering the basal portion of sternum VIII. Sternum VIII forming rectangular subgenital plate, with a distinct longitudinal median cleft, forming a membranous notch that divides the plate into pair of dark, broad, and strongly sclerotized lobes. Paragenital plates small, irregularly subtriangular, located near the middle of the lateral margins of the subgenital plate. Paraprocts short and broad, somewhat semicircular, with obtuse apices. Inner genitalia (Figure 10B) mostly membranous, semicircular, with small, darkly sclerotized structure, connected to the spermathecal ductus.

**Etymology.** The specific epithet ‘gudoushan’ is derived from the Gudoushan Provincial Nature Reserve, the type locality where the holotype was collected. The name is used as a noun in apposition and is gender-neutral.

**Distribution.** China (Guangdong Province).

**Remarks**. The new species differs from other members of the group by the distinctive arrowhead-shaped distal portion of the dorsal sclerite of the epiproct. The female is similar to that of *Amphinemura meizhouensis* Mo, Wang, Yang, Li & Murányi, 2021 in the shape of the subgenital plate, but differs in having a small, darkly sclerotized structure in the vagina, which is absent in *A. meizhouensis* [15].

#### 3.2.4. *Amphinemura prominens* Wang, Wang & Li, sp. nov.

**Figure 11:**
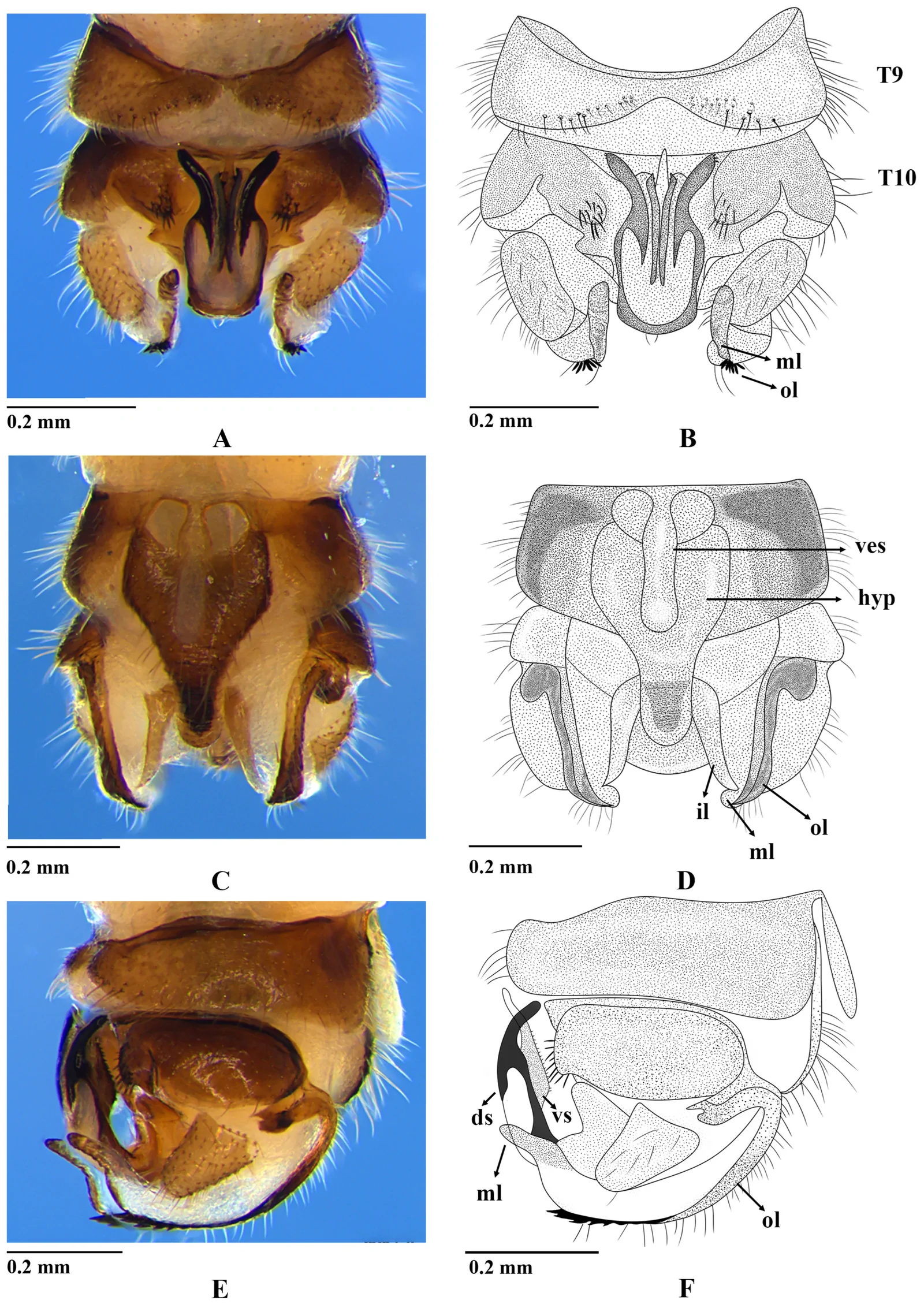
*Amphinemura prominens* sp. nov., male terminalia. (**A**,**B**) Dorsal view; (**C**,**D**) ventral view; (**E**,**F**) lateral view. Abbreviations: T9, tergum 9; T10, tergum 10; ds, dorsal sclerite; vs, ventral sclerite; ves, vesicle; hyp, hypoproct; il, inner lobe; ml, median lobe; ol, outer lobe.

**Figure 12:**
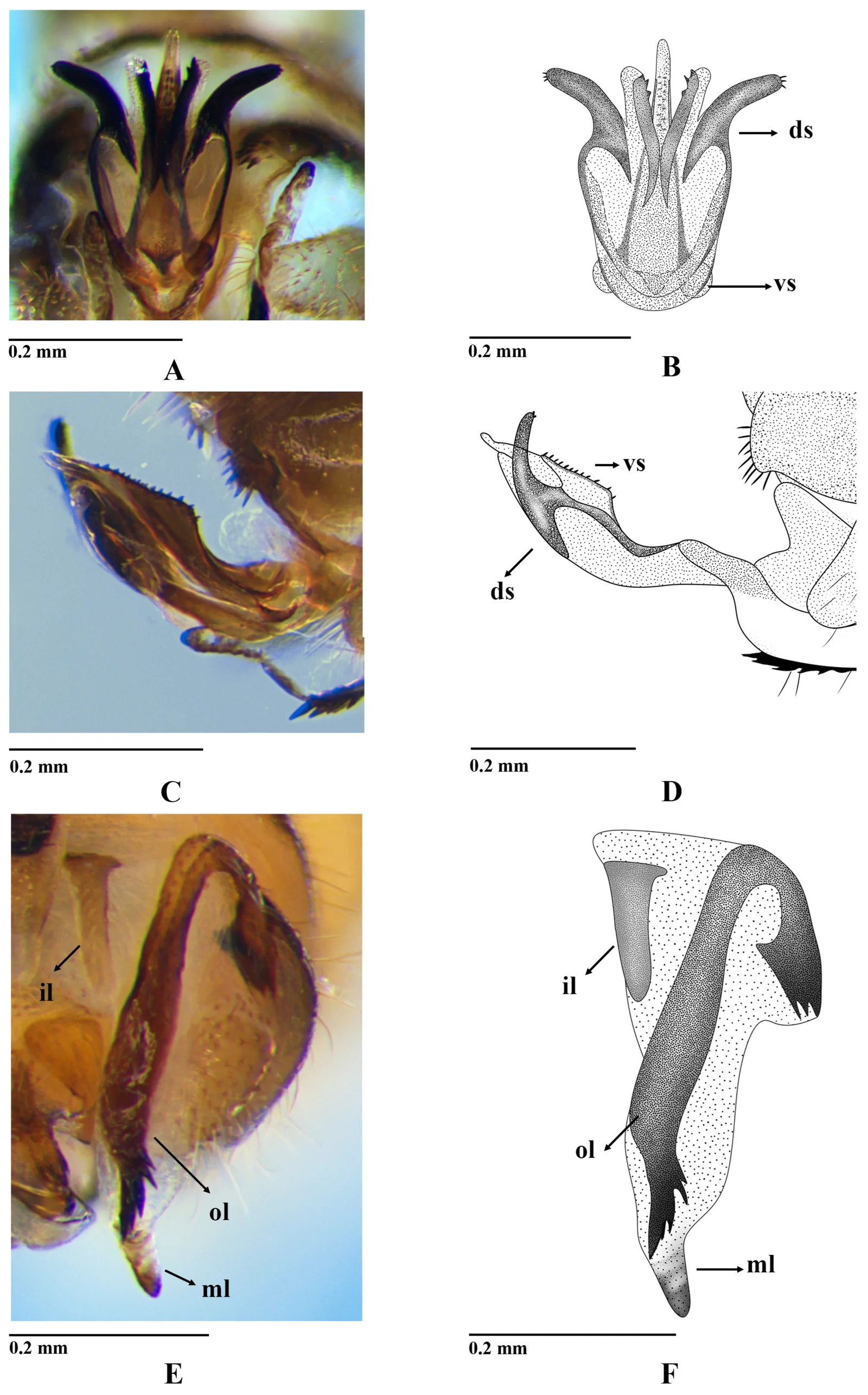
*Amphinemura prominens* sp. nov., male. (**A**–**D**) Epiproct: (**A**,**B**) dorsal view; (**C**,**D**) lateral view; (**E**,**F**) paraproct, caudal view. Abbreviations: ds, dorsal sclerite; vs, ventral sclerite; il, inner lobe; ml, median lobe; ol, outer lobe.

**Figure 13:**
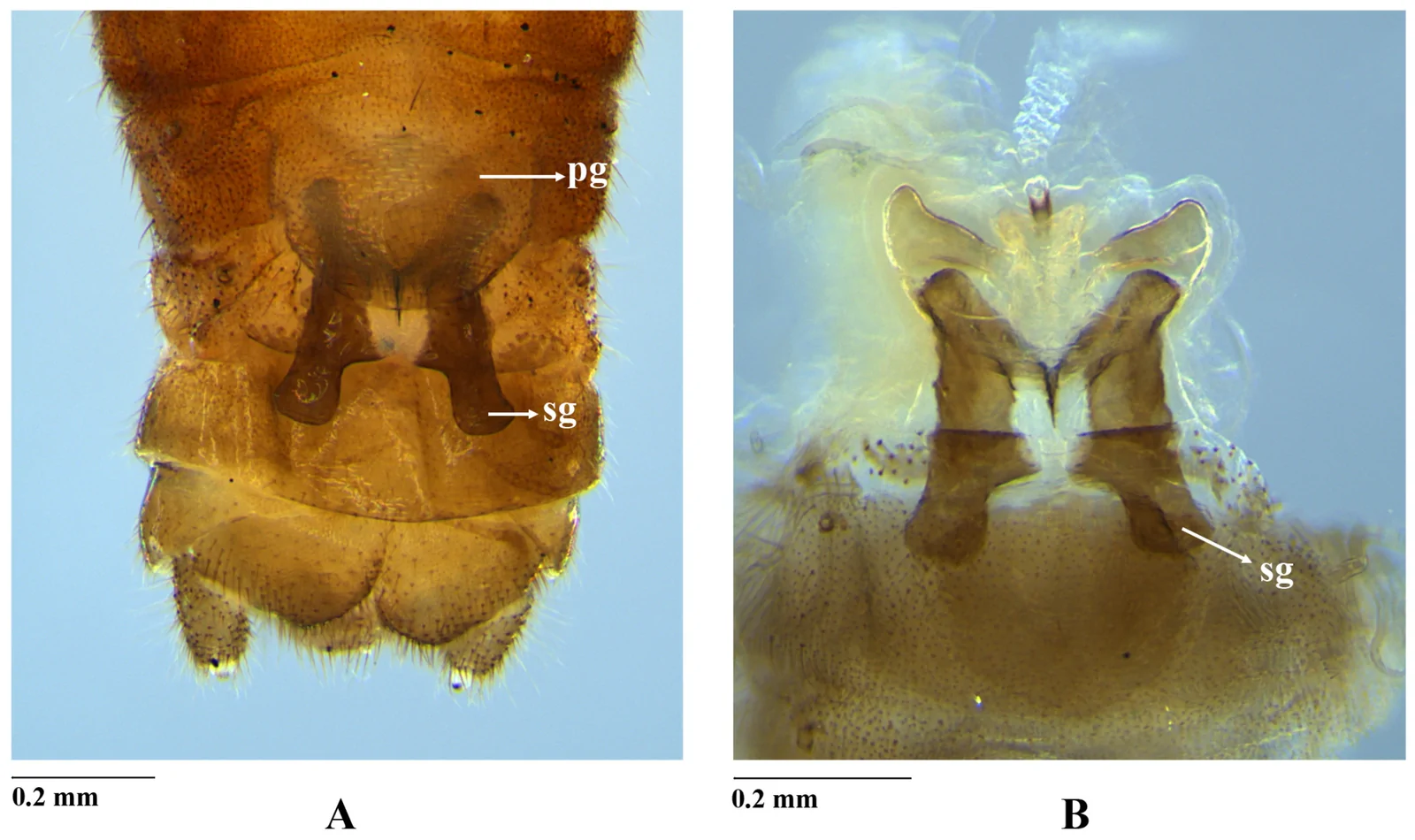
*Amphinemura prominens* sp. nov., female. (**A**) Terminalia after being cleared, ventral view; (**B**) inner genitalia, ventral view. Abbreviations: pg: pregenital plate; sg: subgenital plate.

Chinese common name: 显突倍叉𧐐

(Figure 11, Figure 12 and Figure 13)

**Material examined. Holotype.** ♂ (HIST), China, Guizhou Province, Qiandongnan Miao and Dong Autonomous Prefecture, Leishan County, Leigong Mountain National Nature Reserve, Leigong Mountain Station, Malaise trap, 1297 m, 26°21′44″ N, 108°09′39″ E, 24-VI-2019, Tao Li leg. **Paratypes.** 3♂4♀ (NHMC), China, Guangxi Zhuang Autonomous Region, Liuzhou City, Rongshui County, Wangdong Township, Pingchong Village, Light, 992 m, 25°11′00″ N, 108°43′59″ E, 8-V-2024, Yingying Wang leg.; 1♀ (NHMC), China, Guizhou Province, Qiandongnan Miao and Dong Autonomous Prefecture, Leishan County, Leigong Mountain National Nature Reserve, Leigong Mountain Station, 1595 m, 26°21′44″ N, 108°09′39″ E, 24-VI-2019, Malaise trap, Tao Li leg.

**Diagnosis. ***Amphinemura prominens* is distinguished from congeners in having an epiproct with spiny membranous projections and an unarmed membranous median lobe bearing a finger-like process.

**Description.**

**Adult habitus.** General body brown. Head dark brown; compound eyes black; antennae mostly brown, palpi brown; head slightly wider than pronotum; pronotum subquadrate; legs brown, tarsi darker. Wing membranes subhyaline, veins dark brown. Abdominal segments brown with dark brown terminalia.

**Male**. (Figure 11 and Figure 12). Forewing length 5.8–6.0 mm, hindwing length 4.7–4.9 mm (*n* = 3). Tergum IX (Figure 11A,B) strongly sclerotized, anterior margin concave, with several bristles present along posterior margin. Slender vesicle of sternum IX (Figure 11B,C) claviform, length 3× maximum width, basally and medially slightly constricted. Hypoproct subrectangular basally, basal third slightly swollen and then gradually tapering. Tergum X (Figure 11A,B) strongly sclerotized, presenting membranous concavity below the epiproct, with two groups of black stout spines laterally. Cercus (Figure 11) cylindrical, about 1.5× as long as maximum width. 

Epiproct (Figure 11A,B and Figure 12A–D) about as long as wide, distal portion trifurcate. Dorsal sclerite sclerotized basally, mostly membranous, bearing rows of small spines from middle to apex, pair of slender lateral arms curved outwards extending to a spinous apex. Ventral sclerite dark brown and strongly sclerotized, broad at base, gradually tapering and convergent at tip, forming a triangular ridge with rows of stout black spines along ridge. 

Paraproct (Figure 11B,C and Figure 12E,F) trilobed: inner lobe triangular and sclerotized, with apex obtuse and curved outwards; median lobe membranous, lacking spines, bearing a strongly sclerotized finger-like process apically in ventral view; outer lobe irregular, basal portion darkly sclerotized and distinctly curved downward apically, bearing a row of four to six short, black spines subapically on the ventral face, apex strongly incurved.

**Female** (Figure 13). Forewing length 6.1–6.5 mm, hindwing length 5.5–5.8 mm (*n* = 3). Sternum VII produced into broadly semicircular pregenital plate, covering half the length of Sternum VIII; subgenital plate pale (Figure 13A). Sternum VIII forms sclerotized, rectangular and bicolored subgenital plate, medial notch shallow with a wide light median longitudinal stripe. Paragenital plates paired. Paraprocts brownish, broad at base, narrower at blunt apex (Figure 13). Inner genitalia (Figure 13B) mostly membranous, semicircular, with short anteromedial tubular structure, connected to the spermathecal ductus.

**Etymology.** The specific epithet ‘prominens’ is derived from the Latin ‘prominens’ (prominent), referring to the prominent finger-like process at the apex of the median lobe of the paraproct. The name is used as an adjective.

**Distribution.** Known from Guizhou Province of China and Guangxi Zhuang Autonomous Region.

**Remarks**. The new species is assigned to the *A. sinensis* species group. The new species is similar to *A. xiangae* Zhao & Du, 2022, a species described from Guangdong. These two species share similarities in the structure of the dorsal surface of the epiproct and the shape of the inner lobe of the paraproct. However, *A. prominens* can be easily distinguished from *A. xiangae* by the morphology of the median lobe of the paraproct. In *A. prominens*, the median lobe is lacking spines and bears a strongly sclerotized, finger-like process apically. However, in *A. xiangae*, both the median lobe and the outer lobe of the paraproct each bear a prominent apical spine (see Figure 4 in Zhao et al. [3]). The female is similar to that of *Amphinemura xiangae* Zhao, Du & Rehman, 2022, but the COI data support their separation as distinct species.

## 4. Discussion

The *Amphinemura sinensis* species group is morphologically conservative, and species identification often relies on subtle differences in the male terminalia. The four new species described in this study increase the known diversity of the group to 34 species. They are mainly distinguished by differences in the epiproct and paraprocts, which are also important diagnostic characters in other members of the group. The importance of these structures in species identification is consistent with previous taxonomic studies of the group.

The COI data were generally consistent with morphological identification, with the four new species recovered as distinct clades in the ML tree. However, the ranges of intraspecific and interspecific genetic distances showed a slight overlap. The maximum intraspecific distance (0.016), observed in *A. meizhouensis*, exceeded the minimum interspecific distance (0.013) between *A. chui* and *A. nigritubulata*. Although these two species are morphologically distinguishable, their relatively low COI divergence suggests that genetic distance alone may not be sufficient for species delimitation in the *A. sinensis* species group. Similar overlaps and limitations of COI barcoding have been reported in other insect groups [24,25]. Thus, COI should be considered complementary to morphological evidence rather than used as the sole basis for species delimitation.

The molecular dataset remains limited because several species are represented by only a single COI sequence, restricting the assessment of intraspecific variation. Broader sampling, particularly multiple specimens from different populations, together with additional molecular markers, would provide a stronger basis for evaluating species boundaries within the group.

## Figures and Tables

**Table 1:** A list of specimens sequenced in this study used for phylogenetic analysis, included species studied, Voucher ID, location and GenBank accessions.

Species	Voucher ID	Sex	Province	Accession
*A. prominens* sp. nov.	W284	Female	Guangxi	PZ834979
*A. prominens* sp. nov.	W285	Male	Guangxi	PZ834980
*A. quadrispina* sp. nov.	W286	Female	Guangxi	PZ834981
*A. quadrispina* sp. nov.	W287	Female	Guangxi	PZ834982
*A. quadrispina* sp. nov.	W288	Male	Guangxi	PZ834983
*A. quadrispina* sp. nov.	W290	Male	Guangxi	PZ834984
*A. prominens* sp. nov.	W295	Female	Guangxi	PZ834985
*A. xiangae* Zhao, Du & Rehman, 2022	W304	Male	Guangdong	PZ834986
*A. coronata* sp. nov.	W305	Male	Guizhou	PZ844031
*A. coronata* sp. nov.	W307	Female	Guizhou	PZ834987
*A. maoi* (Wu, 1938)	W315	Male	Hubei	PZ834990
*A. albicauda* Li, Mo, Dong, Yang & Murányi, 2018	W317	Male	Sichuan	PZ834991
*A. albicauda* Li, Mo, Dong, Yang & Murányi, 2018	W318	Male	Shaanxi	PZ834992
*A. oxyacantha* Zhao & Du, 2021	W323	Male	Zhejiang	PZ834993
*A. oxyacantha* Zhao & Du, 2021	W324	Male	Zhejiang	PZ834994
*A. chui* (Wu, 1935)	W333	Male	Fujian	PZ834996
*A. elongata* Li, Yang & Sivec, 2005	W340	Male	Zhejiang	PZ834997
*A. elongata* Li, Yang & Sivec, 2005	W341	Female	Zhejiang	PZ834998
*A. nigritubulata* Li & Yang, 2008	W350	Male	Yunnan	PZ844033
*A. tianmushana* Li & Yang, 2011	W360	Male	Zhejiang	PZ834999
*A. sinensis* (Wu, 1926)	W366	Male	Henan	PZ844032
*A. gudoushan* sp. nov.	W379	Male	Guangdong	PZ835000
*A. gudoushan* sp. nov.	W380	Female	Guangdong	PZ835001
*A. yao* Mo, Yang, Wang & Li, 2017	W382	Female	Guangxi	PZ835002
*A. simifleurdelia* Mo, Wang, Yang, Li & Murányi, 2020	W391	Male	Guangdong	PZ835003
*A. meizhouensis* Mo, Wang, Yang, Li & Murányi, 2021	W397	Female	Guangdong	PZ835004
*A. meizhouensis* Mo, Wang, Yang, Li & Murányi, 2021	W398	Female	Guangdong	PZ835005

## Data Availability

The original contributions presented in this study are included in the article/Supplementary Material. Further inquiries can be directed to the corresponding author(s).

## Supplementary Materials

The following supporting information can be downloaded at: https://www.mdpi.com/article/10.3390/insects17090909/s1, Table S1: Pairwise genetic distances among COI sequences of the Amphinemura sinensis species group used in the phylogenetic analysis, calculated using the Kimura 2-parameter (K2P) model.
